# Dual Antiplatelet Therapy vs. Single Antiplatelet Therapy After Transcatheter Aortic Valve Replacement: An Updated Systematic Review and Meta-Analysis

**DOI:** 10.3389/fcvm.2021.679703

**Published:** 2021-06-21

**Authors:** Yipeng Zhang, Lan Shen, Wentao Yang, Ben He

**Affiliations:** ^1^Department of Cardiology, Shanghai Chest Hospital, Shanghai Jiao Tong University, Shanghai, China; ^2^Shanghai Jiao Tong University School of Medicine, Shanghai, China

**Keywords:** transcatheter aortic valve replacement, dual antiplatelet therapy, single antiplatelet therapy, bleeding, mortality, myocardial infarction, stroke, meta-analysis

## Abstract

**Background:** Although mainstream guidelines recommend dual antiplatelet therapy (DAPT) with aspirin and clopidogrel in patients following transcatheter aortic valve replacement (TAVR), it is not evidence-based. We aim to investigate the safety and efficacy of DAPT vs. single antiplatelet therapy (SAPT) after TAVR, and review updated evidence.

**Methods:** We systematically searched PubMed, Embase, and Cochrane for studies comparing DAPT to SAPT after TAVR from inception to November 30, 2020. The primary outcome was major adverse cardiac and cerebrovascular events, including all-cause mortality, cardiovascular death, myocardial infarction (MI), stroke, and major or life-threatening bleeding (LTB). Subgroup analysis was performed according to study type (randomized control trials vs. observational studies) using a fixed-effects model. The quality of evidence was assessed by two scoring systems and GRADE (Grading of Recommendations Assessment, Development, and Evaluation).

**Results:** Twelve studies of 20,766 patients were included in our meta-analysis. Compared with SAPT, DAPT was associated with an increased risk for combined life threatening and major bleeding [OR 1.73 (1.19–2.51), *p* = 0.004] after TAVR. Such a difference was largely driven by major bleeding [OR 2.29 (1.68–3.11), *p* < 0.001]. There were no significant differences on major adverse cardiovascular events (MACE) [OR 1.19 (0.99–1.44), *p* = 0.07], cardiovascular mortality [OR 1.46 (0.93–2.30), *p* = 0.10], and stroke [OR 0.97 (0.80–1.16), *p* = 0.71].

**Conclusions:** Compared with SAPT, post-TAVR DAPT was associated with increased risks of major or life-threatening bleeding without additional benefits of reducing thrombotic events. Future guidelines for post-TAVR antiplatelet strategy are expected to be updated as new high-quality evidence emerges.

**Systematic Review Registration:** PROSPERO, Identifier: CRD42021230075.

## Introduction

Transcatheter aortic valve replacement (TAVR) has emerged as an effective and alternative therapeutic strategy for patients with severe symptomatic aortic valve stenosis, especially for those with intermediate or high surgical risk of surgical aortic valve replacement (SAVR) (1, 2). Despite its high procedure success rate and non-inferiority efficacy to SAVR, TAVR has been associated with severe major adverse cardiac and cerebrovascular complications such as mortality, myocardial infarction (MI), major stroke, life-threatening bleeding (LTB), and major vascular complication (3–5).

To reduce the risk of embolization events, the American College of Cardiology (ACC)/American Heart Association (AHA) 2020 guidelines recommend the use of dual antiplatelet therapy (DAPT) with clopidogrel and aspirin for the first 3–6 months after TAVR in patients who are at low risk of bleeding (1). Although intensive antiplatelet therapy with DAPT theoretically decreases the potential risk of thrombotic events, there comes a substantially increased risk of bleeding compared with single antiplatelet therapy (SAPT). The optimal antiplatelet regimen remains unclear. Considering the controversial findings in existing meta-analyses and availability of the latest randomized clinical trials (RCTs), we conducted an updated meta-analysis of RCT and observational studies to review the risk and benefit of the two post-TAVR antiplatelet therapies.

## Methods

### Search Strategy and Selection Criteria

This meta-analysis is reported in accordance with the Preferred Reporting Items for Systematic Reviews and Meta-analysis (PRISMA) statement (6) and was registered at the International Prospective Register of Systematic Reviews (number CRD42021230075).

We selected randomized control trials and observational studies published from inception to November 30, 2020, by searching Embase, PubMed, Cochrane, Web of Knowledge, and ClinicalTrials.gov. No language restrictions were applied. We used the following terms relating to TAVR (transcatheter aortic valve replacement, transcatheter aortic valve implantation, TAVI, TAVR) and intervention (DAPT, SAPT, dual antiplatelet therapy, single antiplatelet therapy, antithrombotic, antiplatelet, aspirin, clopidogrel) as keywords and mesh terms. The related articles and reference lists of studies were also screened manually to identify additional relevant publications.

### Study Selection and Data Extraction

Studies were eligible for inclusion if they met the following criteria: (1) RCTs or observational studies; (2) patients with aortic stenosis or regurgitation undergoing TAVR; (3) comparative study of different antithrombotic strategies post-TAVR including DAPT, SAPT; (4) reported at least one adverse event (such as death, myocardial infarction, stroke, and bleeding); and (5) articles published in English.

Study titles and abstracts were reviewed for eligibility by two independent investigators (YZ and WY), and studies that satisfied the inclusion criteria were retrieved for full-text assessment. Two investigators (YZ and WY) carried out data extraction independently, with disagreements resolved by a third investigator (BH). We extracted the following data from each selected study: (1) basic information of the study including first author, year of publication, duration of follow-up, study type, and inclusion and exclusion criteria; (2) baseline data of enrolled patients; (3) detailed cardiovascular clinical outcome. Data from the propensity score-matched cohort were preferentially abstracted if available.

### Outcomes

The main outcomes were major adverse cardiac and cerebrovascular events, including all-cause mortality, cardiovascular death, myocardial infarction, stroke, and major or life-threatening bleeding. MACE was defined as a composite of all-cause mortality, major stroke, and MI. All clinical events were defined according to the Valve Academic Research Consortium-2 (VARC-2) (7). Both in-hospital and discharge follow-up endpoint events were accounted for in the analysis. The longest reported follow-up duration was used to analyze the main endpoints when multiple follow-up durations existed. Subgroup analysis was performed according to study type (RCTs vs. observational studies).

### Statistical analysis

This meta-analysis was performed with RevMan 5.3 (Review Manager [RevMan] Version 5.3. Copenhagen: The Nordic Cochrane Center, The Cochrane Collaboration; 2014). The pooled odds ratios (OR) were calculated by combining individual studies using a fixed-effects model when the heterogeneity was not evident. Total ORs were calculated and reported with 95% confidence intervals (CI). Heterogeneity was tested by the Chi-squared test and the *I*^2^-test. Heterogeneity was identified when the *P* < 0.1 or *I*^2^ > 50%. Funnel plots were constructed to assess publication bias. Sensitivity analysis was performed by excluding a single study at a time.

## Results

### Search Results

**Figure 1:**
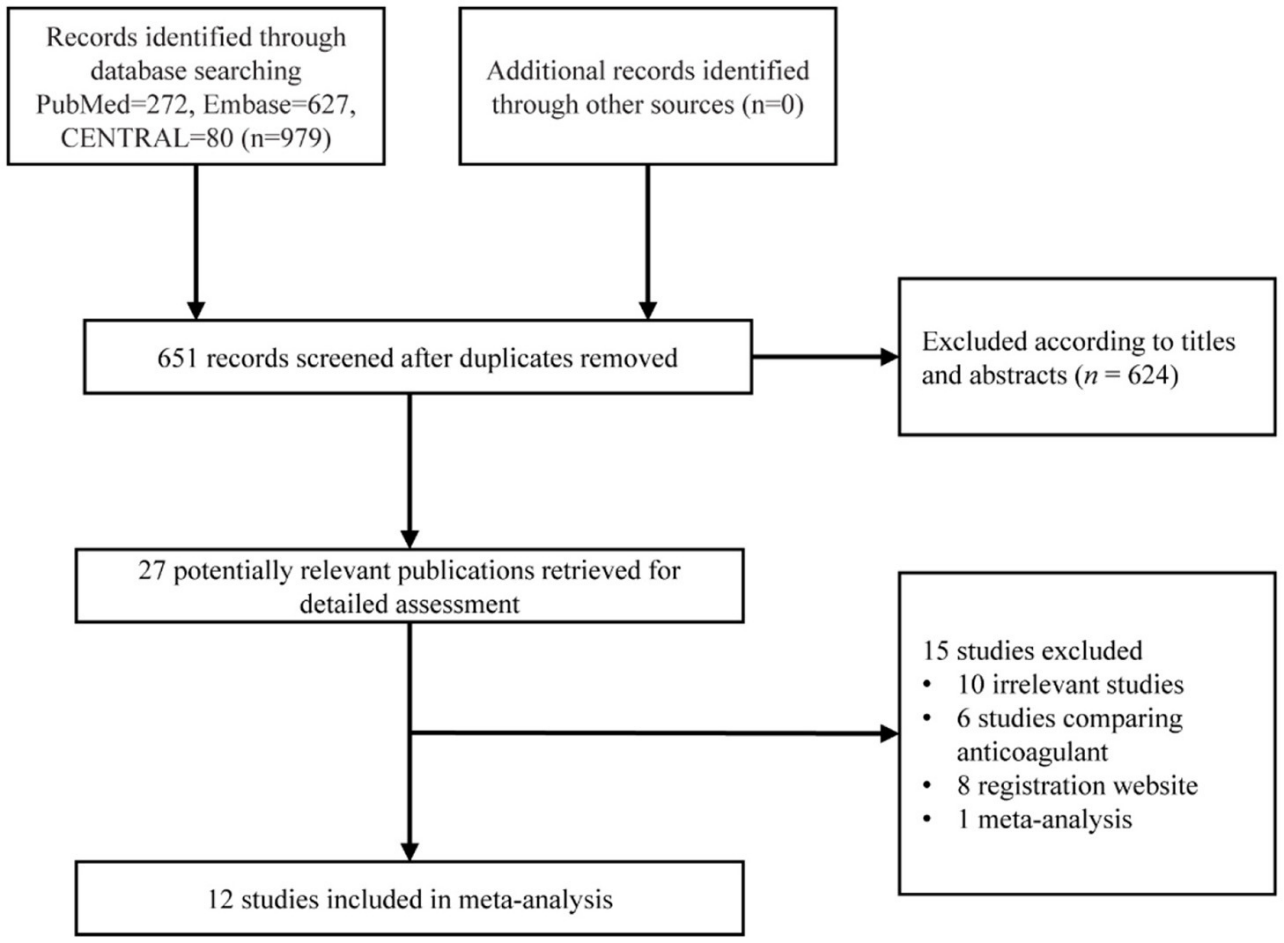
Study flow chart of the search strategy and selection process for inclusion.

A detailed study selection process is shown in Figure 1. In short, 979 articles were collected based on our search strategy, of which 328 were duplicated articles. Further, 624 were excluded after screening the titles and abstracts. The full text was reviewed for the remaining 27 articles, and of those, 6 were studies comparing anticoagulant strategy, 8 were registration websites, 10 were irrelevant articles, and 1 was a meta-analysis. Finally, 12 studies with 20,766 patients were enrolled for our meta-analysis (8–19).

### Characteristics of Included Studies

Of the 12 included studies, four were RCTs (8–11), and eight were observational studies (12–19). Three observational studies applied propensity score-matched analyses (13, 15, 16). All except three studies were small sample studies. Only three studies had more than 500 patients (11, 15, 18), and especially one incorporating 16,694 patients had a large weight and substantial impact on the meta-analysis (18). As for the longest follow-up time with detailed outcomes available, one study had only in-hospital outcomes (14), three studies had 30 day outcomes (9, 12, 13), one study had 3 month outcomes (10), two studies had 6 month outcomes (8, 9), four studies had 1-year outcomes (11, 16–18). Five of the included studies used SAPIEN series devices with a large percentage (9, 10, 14–16). **One** study had only detailed bleeding outcomes available (14). Two studies had unusual endpoints with bleeding and prosthetic valve dysfunction, respectively (11, 15). Detailed characteristics of the eligible studies are outlined in Table 1.

**Table 1:** Characteristics of included studies.

**Study**	**Ussia et al. (8) (*N* = 79)**	**Stabile et al. (9) (*N* = 120)**	**Rodés-Cabau et al. (10) (*N* = 222)**	**Brouwer et al. (11) (*N* = 665)**	**Poliacikova et al. (12) (*N* = 114)**	**Durand et al. (13) (*N* = 182)**
Type	RCT	RCT	RCT	RCT	Prospective observational study	Prospective observational study
Enrollment date	May 2009–August 2010	April 2010–April 2011	March 2012–February 2017	February 2014–2018	December 2007–June 2012	January 2010–December 2011
Inclusion criteria	1. Severe symptomatic AS with AVA <1 cm^2^ 2. Refused for standard AV replacement	1. Severe AS: echocardiographically derived AVA <0.8 cm^2^ (or AVA index <0.5 cm^2^/m^2^) and mean AVG > 40 mm Hg or peak jet velocity >4.0 m/s 2. Cardiac symptoms: NYHA functional class II, syncope 3. High surgical risk: predicted operative mortality 15% or STS score 10%	Patients with clinical indications for TAVR with a balloon-expandable Edwards SAPIEN XT or SAPIEN 3 valve	1. TAVI population consisting of both high-risk surgical and inoperable patients 2. Patient has provided written informed consent	1. Severe symptomatic AS with AVA observational <1 cm^2^ 2. Refused for standard AV replacement	1. Patients with symptomatic severe AS who were not candidates for surgical AV replacement because of coexisting illness 2. AVA <0.8 cm^2^, mean aortic gradient ≥40 mm Hg or a peak aortic jet velocity ≥4.0 m/s 3. NYHA functional class II, III, or IV
Exclusion criteria	1. Vascular disease that precluded access 2. Severe deformation of the chest 3. Intracardiac thrombus 4. Unprotected stenosis of the left main coronary artery not amenable to PCI 5. MI within 7 days 6. Prosthetic heart valve 7. Active infection 8. Leukopenia 9. Coagulopathy 10. Active bleeding 11. Acute anemia (Hb <9 mg/dL) 12. Aorta could not be fully dilated with a 23-mm aortic valvuloplasty balloon 13. Aortic annulus size <19 or >24 mm 14. Liver cirrhosis 15. Recurrent pulmonary embolism 16. Porcelain aorta 17. Respiratory failure 18. History of radiotherapy to mediastinum 19. Severe connective tissue disease 20. Previous PCI or MI requiring DAPT 21. Need for oral anticoagulation therapy 22. Allergy or intolerance to study drugs	1. Aortic annular diameter on echocardiography <18 or >25 mm 2. Aortic dissection, or iliac-femoral dimensions or disease precluding safe sheath insertion 3. Untreated coronary artery disease requiring revascularization 4. Severe aortic regurgitation or mitral regurgitation (>3t:J), or prosthetic valve 5. Acute myocardial infarction within 1 month 6. Upper gastrointestinal bleeding within 3 months 7. Cerebrovascular accident or transient ischemic attack within 6 months 8. Any cardiac procedure, other than balloon aortic valvuloplasty, within 1 or 6 months of drug-eluting stent placement 9. Indication for oral anticoagulation therapy (i.e., atrial fibirllation) 10. Aspirin intolerance/allergy 11. Thienopyridine intolerance/allergy	1. Need for chronic anticoagulation treatment 2. Major bleeding within the 3 months before the TAVR procedure 3. Prior intracranial bleeding, drug eluting stent implantation within the year before the TAVR procedure 4. Allergy to clopidogrel and/or aspirin	1. Need for long-term OAC 2. Drug-eluting stent implantation within 3 months prior to TAVI procedure 3. Bare-metal stent implantation within 1 month prior to TAVI procedure 4. Allergy or intolerance to aspirin or clopidogrel	1. Vascular disease that precluded access 2. Severe deformation of the chest 3. Intracardiac thrombus 4. Unprotected stenosis of the left main coronary artery not amenable to PCI 5. MI within 7 days 6. Prosthetic heart valve 7. Active infection 8. Leukopenia 9. Coagulopathy 10. Active bleeding 11. Acute anemia (Hb <9 mg/dL) 12. Aorta could not be fully dilated with a 23 mm aortic valvuloplasty balloon 13. Aortic annulus size <19 or >24 mm 14. Liver cirrhosis 15. Recurrent pulmonary embolism 16. Porcelain aorta 17. Respiratory failure 18. History of radiotherapy to mediastinum 19. Severe connective tissue disease 20. Previous PCI or MI requiring DAPT 21. Need for oral anticoagulation therapy 22. Allergy or intolerance to study drugs	Pretreatment with DAPT
Medication DAPT group	1. Aspirin 10.0 mg/day 2. Clopidogrel 300 mg loading then 75 mg/day for 3 months	1. Aspirin 80 mg/day 2. Clopidogrel 75 mg/day or ticlopidine 500 mg BID for 6 months	1. Aspirin or acetylsalicylic acid (80–100 mg/day) for at least 6 months 2. Clopidogrel (75 mg/day) for 3 months	Aspirin at a dose of 80 to 100 mg daily plus clopidogrel at a dose of 75 mg daily for 3 months	1. Aspirin 75 mg/day 2. Clopidogrel 300 mg loading then 75 mg/day for 6 months	1. Aspirin 75 mg/day 2. Clopidogrel 300 mg loading then clopidogrel 75 mg/day; duration 1–6 months
Medication SAPT group	Aspirin 100 mg/day; duration 3 months	Aspirin 75–160 mg/day for 6 months	Aspirin or acetylsalicylic acid (80–100 mg/day) for at least 6 months	Aspirin 80–100 mg/d for 3 months	Aspirin 100 mg/day for 3 months	Aspirin 75 mg/day or clopidogrel 75 mg/day (without clopidogrel loading dose) for 1–6 months
Follow-up included	6 months	30 days, 6 months	1, 2, 3 months	1, 2, 6 months, 1 year	30 days	30 days
Primary endpoint	Composite of death from any cause, MI, major stroke, urgent or emergency conversion to surgery, and LTB	Composite of major stroke, acute coronary event, all-cause mortality, major, and lethal bleeding	Rate of death, MI, ischemic stroke or TIA, or major or life-threatening bleeding at 3 month follow-up	all bleeding (including minor, major, and life-threatening or disabling bleeding) and non-procedure-related bleeding over a period of 12 months	MACE (combined endpoint of all-cause mortality, ACS or stroke) and NACE (combined endpoint of all-cause mortality, ACS, stroke, or major bleeding)	Combination of mortality, major stroke, LTB, MI, and major vascular complications at 30 days
TAVR device	CoreValve	SAPIEN	SAPIEN XT or SAPIEN 3	NA	CoreValve	SAPIEN, or CoreValve
**Study**	**Czerwińska-Jelonkiewicz et al**. **(****14****)** **(*****N*** **=** **476)**	**D'Ascenzo et al**. **(****15****)** **(*****N*** **=** **1,210)**	**Ichibori et al**. **(****16****)** **(*****N*** **=** **88)**	**Mangieri et al**. **(****17****)** **(*****N*** **=** **439)**	**Sherwood et al**. **(****18****)** **(*****N*** **=** **16,694)**	**Munoz-Garcia et al**. **(****19****)** **(*****N*** **=** **477)**
Type	Retrospective observational study	Retrospective observational study	Retrospective observational study	Retrospective observational study	Retrospective observational study	Observational study (Conference abstract)
Enrollment date	April 2013–2014	January 2007–December 2012	October 2009–January 2015	January 2009–May 2015	October 2011–June 2016	April 2008–December 201
Inclusion criteria	All patients who underwent TAVI in the participating centers between 2013 and 2014 were included	All patients undergoing balloon expandable TAVR who are not on oral anticoagulant therapy with vitamin K antagonists	Patients who underwent TAVR using balloon expandable aortic valves because of severe aortic valve stenosis	All patients with severe aortic stenosis underwent intervention through transfemoral approach	Patients without pre-operative atrial fibrillation from STS/ACC TVT registry	NA
Exclusion criteria	NA	Patients on oral anticoagulant therapy with vitamin K antagonists	Patients who had indications for oral anticoagulant therapy before TAVI	1. Patients on anticoagulant therapy (either as monotherapy or with an antiplatelet agent) 2. Patients who died during index hospitalization 3. Patients who underwent TAVR discharged without any antiplatelet/anticoagulant therapy because of a prohibitive risk of bleeding 4. TAVR procedure not performed through a transfemoral route	1. Patients with atrial fibrillation 2. Patients with non-transfemoral access 3. Patients who had in-hospital death and those with missing data on discharge medications and procedural complications 4. Patients discharged without any antiplatelet therapy	NA
Medication DAPT group	Aspirin and clopidogrel	1. Aspirin 2. Clopidogrel for at least 6 months	1. Lifelong low-dose aspirin 100 mg daily for 6 months 2. Ticlopidine 200 mg or clopidogrel 75 mg daily for 6 months	Aspirin (75/100/150 mg/day) clopidogrel (75 mg/day) within 3–6 months	Aspirin and clopidogrel	NA
Medication SAPT group	Aspirin	Aspirin for at least 6 months	Lifelong low-dose aspirin 100 mg daily for 6 months	Either ASA or clopidogrel for 3–6 months	Aspirin	NA
Follow-up included	In-hospital duration	30 days	1 year	1 year	1 year	NA
Primary endpoint	Single, in-hospital safety and efficacy events such as: severe bleeding, vascular complications, thromboembolic events, myocardial infarction and 30-day, all-cause mortality	Prosthetic heart valve dysfunction at follow-up (defined as diagnosis of aortic valve <1.2 cm^2^, an increase of medium gradient of more than 20 mmHg and a peak velocity of more than 3 m/s, excluding aortic valve regurgitation)	All-cause death, non-fatal myocardial infarction, non-fatal stroke, and major or life-threatening bleeding complications	Composite of all-cause mortality, myocardial infarction, cerebrovascular events, major bleeding requiring hospitalization, and valve thrombosis	Composite of stroke, all 1 year cause mortality and major bleeding	NA
TAVR device	Sapien/Sapien XT or CoreValve	Sapien and Sapien XT	SAPIEN or SAPIEN XT	SAPIEN XT or SAPIEN 3	All available	NA

*AS, aortic stenosis; AVA, aortic valve area; AV, aortic valve; PCI, percutaneous coronary intervention; TIA, transient ischemic attacks; MACE, major adverse cardiovascular and cerebrovascular events*.

### Patient and Procedure Characteristics

Patient baseline data and procedure characteristics are shown in Table 2. Patients included in the two groups were similarly elderly (83.78 y) and tended to have multiple comorbidities, including hypertension (79.1%), DM (26.6%), and CKD (10.1%). The DAPT group was more likely to undergo previous PCI, MI, and coronary artery disease compared with SAPT mainly due to selection bias in observational studies. Patients included had a mean EuroSCORE of 21.83% and a mean Society of Thoracic Surgeons (STS) score of 6.00%, with a mean aortic valve area of 0.58 cm^2^ and a mean aortic gradient of 50.48 mmHg.

Table 2Baseline characteristics of the included patients and procedure.

**Table:** 

**Study**	**Ussia et al**. **(****8****)**		**Stabile et al**. **(****9****)**		**Rodés-Cabau et al**. **(****10****)**		**Brouwer et al**. **(****11****)**		**Poliacikova et al**. **(****12****)**		**Durand et al**. **(****13****)**	
	**DAPT (40)**	**SAPT (39)**	**DAPT (60)**	**SAPT (60)**	**DAPT (111)**	**SAPT (111)**	**DAPT (334)**	**SAPT (331)**	**DAPT (58)**	**SAPT (91)**	**DAPT (128)**	**SAPT (164)**
Age (y)	80 ± 6	81 ± 4	80.2 ± 5.7	81.1 ± 4.8	79 ± 9	79 ± 9	79.5 ± 6.4	80.4 ± 6.2	81.6 ± 6.3	82 ± 6.9	84.6 ± 5.8	82.7 ± 6.3
Female	20 (50.0)	23 (59.0)	44 (73.3)	36 (60.0)	41 (36.9)	52 (46.8)	NA	NA	26 (44.8)	42 (46.2)	78 (60.9)	74 (45.1)
Hypertension	35 (87.5%)	31 (79.5%)	57 (95.0%)	57 (95.0%)	86 (77.5%)	87	255 (76.3%)	243 (73.4%)	NA	NA	90 (70.3%)	116 (70.7%)
Diabetes	13 (32.5%)	8 (20.5%)	15 (25.0%)	17 (28.3%)	41 (36.9%)	36	85 (25.4%)	78 (23.6%)	16 (27.6%)	16 (17.6%)	30 (23.4%)	40 (24.4%)
CKD	NA	NA	NA	NA	NA	NA	NA	NA	3 (5.2%)	5 (5.5%)	11 (8.6%)	12 (7.3%)
Stroke	2 (5.0%)	4 (10.3%)	NA	NA	NA	NA	12 (3.6%)	18 (5.4%)	NA	NA	12 (9.4%)	13 (7.9%)
Atrial fibrillation	4 (10.0%)	6 (15.4%)	NA	NA	NA	NA	NA	NA	16 (27.6%)	10 (11.0%)	45 (35.2%)	37 (22.6%)
NYHAIII, IV	26 (65.0%)	23 (59.0%)	54 (90.0%)	53 (88.3%)	NA	NA	NA	NA	NA	NA	99 (77.3%)	131 (79.9%)
EF (%)	51 ± 12	54 ± 8	52.4 ± 14.4	51.3 ± 11.0	55 ± 12	54 ± 13	NA	NA	NA	NA	60.5 ± 14.0	54.4 ± 13.6
CAD	NA	NA	NA	NA	NA	NA	138 (41.3%)	134 (40.5%)	37 (63.8%)	50 (54.9%)	39 (30.5%)	82 (50.0%)
MI	7 (17.5%)	4 (10.3%)	NA	NA	26 (23.4%)	20	31 (9.3%)	28 (8.5%)	NA	NA	14 (10.9%)	12 (7.3%)
PCI	12 (30.0%)	9 (23.1%)	NA	NA	NA	NA	NA	NA	16 (27.6%)	20 (22.0%)	NA	NA
CABG	2 (5.0%)	4 (10.3%)	NA	NA	39 (35.1%)	42	65 (19.5%)	61 (18.4%)	17 (29.3%)	19 (20.9%)	10 (7.8%)	30 (18.3%)
AV surgery	24 (60.0%)	18 (46.2%)	NA	NA	NA	NA	20 (6.0%)	23 (6.9%)	NA	NA	NA	NA
Pacemaker	4 (10.0%)	1 (2.6%)	NA	NA	NA	NA	NA	NA	NA	NA	10 (7.8%)	18 (11.0%)
AV area (cm^2^)	0.6 ± 0.2	0.6 ± 0.3	NA	NA	0.42 ± 0.13	0.4 ± 0.11	NA	NA	0.67 ± 0.17	0.71 ± 0.22	0.63 ± 0.14	0.61 ± 0.16
AV mean gradient (mmHg)	52 ± 6	57 ± 18	59.4 ± 15.4	63.6	43 ± 16	43 ± 15	NA	NA	NA	NA	48.3 ± 19.5	50.1 ± 15.2
STS (%)	8 ± 5	7 ± 3	9.7 ± 5.1	10.4	6.2 ± 4.4	6.4 ± 4.6	NA	NA	NA	NA	6.9 ± 4	7.4 ± 6.1
EuroScore (%)	23 ± 15	21 ± 16	23.34 ± 8.15	81.1	NA	NA	NA	NA	NA	NA	20.2 ± 11.6	20 ± 12.4
Transfemoral	38 (95.0%)	39 (100.0%)	NA	NA	80 (72.1%)	73	NA	NA	NA	NA	98 (76.6%)	138 (84.1%)
CoreValve	40 (100.0%)	39 (100.0%)	0	0	0	0	NA	NA	NA	NA	0	54 (32.9%)
SAPIEN	0	0	60 (100.0%)	60 (100.0%)	111 (100.0%)	111 (100.0%)	NA	NA	NA	NA	128 (100.0%)	110 (67.1%)

**Table:** 

**Study**	**Czerwińska-Jelonkiewicz et al**. **(****14****)**		**D'Ascenzo et al**. **(****15****)**		**Ichibori et al**. **(****16****)**		**Mangieri et al**. **(****17****)**		**Sherwood et al**. **(****18****)**	
	**DAPT (102)**	**SAPT (102)**	**DAPT (605)**	**SAPT (605)**	**DAPT (66)**	**SAPT (78)**	**DAPT (331)**	**SAPT (108)**	**DAPT (13,546)**	**SAPT (3,148)**
Age (y)	77.9 ± 7.15	78.8 ± 7.55	81 ± 5	81 ± 4	84 ± 6	83 ± 6	82.9 ± 8.2	84.3 ± 7.1	84 ± 5	84 ± 5
Female	NA	NA	336 (55.5)	349 (57.7)	NA	NA	214 (64.7)	62 (57.4)	6,532 (48.2)	1,560 (49.6)
Hypertension	95 (93.1%)	93 (91.2%)	467 (77.2%)	495 (81.8%)	NA	NA	260 (78.5%)	91 (84.3%)	NA	NA
Diabetes	41 (40.2%)	33 (32.4%)	159 (26.3%)	154 (25.5%)	22 (33.3%)	24 (30.8%)	89 (26.9%)	19 (17.6%)	NA	NA
CKD	NA	NA	NA	NA	NA	NA	NA	NA	1,159 (8.6%)	332 (10.5%)
Stroke	5 (4.9%)	5 (4.9%)	NA	NA	13 (19.7%)	22 (28.2%)	48 (14.5%)	13 (12.0%)	1,386 (10.2%)	307 (9.8%)
Atrial fibrillation	20 (19.6%)	14 (13.7%)	75 (12.4%)	54 (8.9%)	NA	NA	54 (16.3%)	22 (20.4%)	NA	NA
NYHAIII, IV	NA	NA	NA	NA	44 (66.7%)	37 (47.4%)	181 (54.7%)	53 (49.1%)	10,626 (78.4%)	2,448 (77.8%)
EF (%)	NA	NA	52 ± 12	51 ± 9	NA	NA	52.6 ± 12.9	51.6 ± 13.4	NA	NA
CAD	NA	NA	226 (37.4%)	224 (37.0%)	35 (53.0%)	29 (37.2%)	NA	NA	8,691 (64.2%)	1,664 (52.9%)
MI	12 (11.8%)	15 (14.7%)	118 (19.5%)	103 (17.0%)	NA	NA	63 (19.0%)	13 (12.0%)	NA	NA
PCI	14 (13.7%)	7 (6.9%)	NA	NA	25 (37.9%)	11 (14.1%)	NA	NA	NA	NA
CABG	NA	NA	NA	NA	10 (15.2%)	10 (12.8%)	70 (21.1%)	20 (18.5%)	NA	NA
AV surgery	NA	NA	55 (9.1%)	42 (6.9%)	NA	NA	12 (3.6%)	7 (6.5%)	3,198 (23.6%)	812 (25.8%)
Pacemaker	NA	NA	NA	NA	NA	NA	18 (5.4%)	9 (8.3%)	NA	NA
AV area (cm^2^)	NA	NA	NA	NA	0.62 ± 0.17	0.7 ± 0.19	NA	NA	NA	NA
AV mean gradient (mmHg)	NA	NA	NA	NA	56 ± 19	50.5 ± 17	NA	NA	NA	NA
STS (%)	9.69 ± 5.27	10.34 ± 6.68	8 ± 7	8 ± 6	12.2 ± 12.7	10.7 ± 7.4	7.2 ± 6.6	7.9 ± 6.7	5.7 ± 2.5	5.5 ± 2.5
EuroScore (%)	20.11 ± 14.64	18.62 ± 14.57	21 ± 14	19 ± 13	25.5 ± 18.5	24.2 ± 15.6	19.4 ± 13	21.3 ± 17.2	NA	NA
Transfemoral	NA	NA	409 (67.6%)	379 (62.6%)	NA	NA	331 (100.0%)	108 (100.0%)	NA	NA
CoreValve	NA	NA	0	0	NA	NA	NA	NA	NA	NA
SAPIEN*	NA	NA	605 (100.0%)	605 (100.0%)	66 (100.0%)	78 (100.0%)	158 (47.7%)	53 (49.1%)	NA	NA

DAPT, dual antiplatelet therapy; SAPT, single antiplatelet therapy; CKD, chronic kidney disease; AF, atrial fibrillation; NYHA, New York Heart Association; EF, ejection fraction; CAD, coronary artery disease; MI, myocardial infarction; PCI, percutaneous coronary intervention; CABG, coronary artery bypass grafting; AV, aortic valve; STS, society of thoracic surgeons; SAPIEN*,SAPIEN series valve including SAPIEN, SAPIEN-XT, SAPIEN-3.

### Quality Assessment

**Figure 2:**
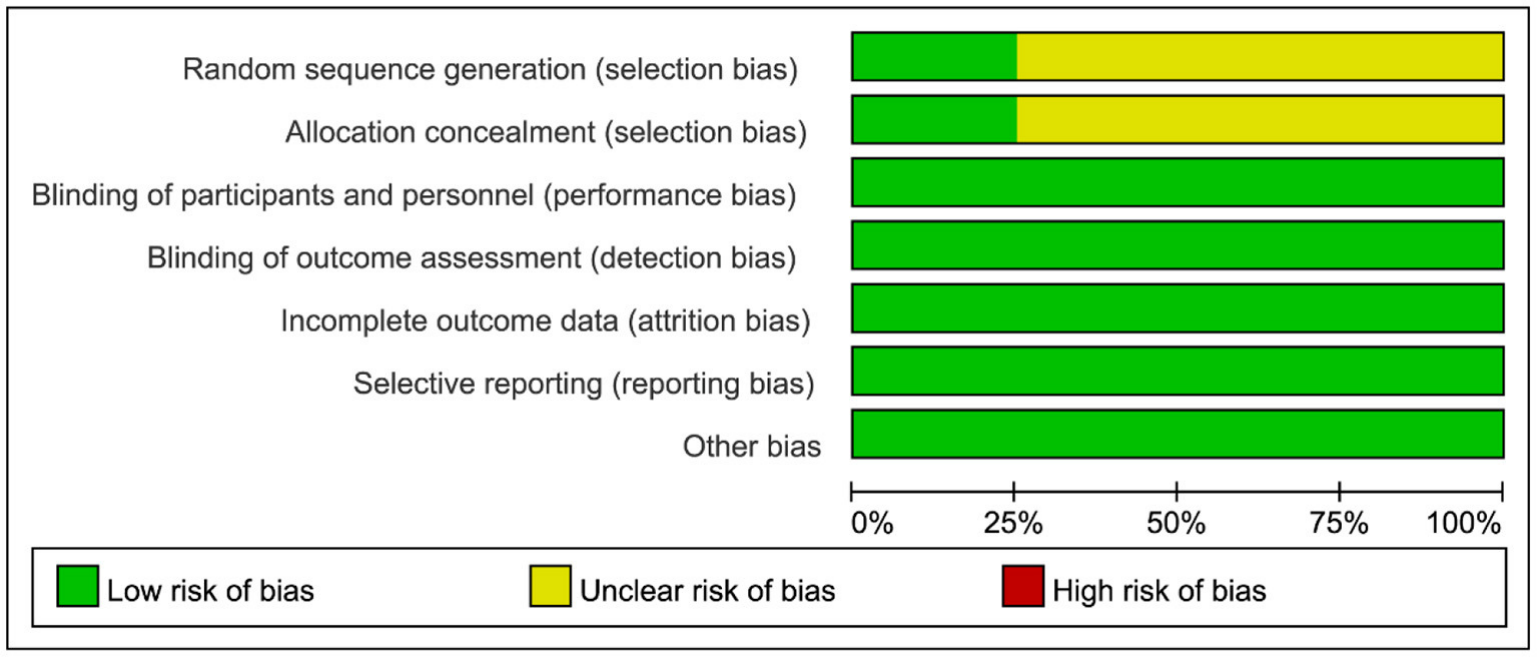
Cochrane Collaboration Risk of Bias Graph of assessment of the quality of RCTs.

The quality of RCTs was assessed by the Cochrane Collaboration Risk of Bias Tool, as illustrated in detail in Figure 2. Observational studies were assessed by the Newcastle-Ottawa Quality Assessment Scale, as shown in Table 3. All the RCTs and seven of the observational studies were considered to be high quality (8–18).

**Table 3:** Assessment of observational studies by the Newcastle-Ottawa quality assessment scale.

	**Poliacikova et al. (12)**	**Durand et al. (13)**	**Czerwińska-Jelonkiewicz et al. (14)**	**D'Ascenzo et al. (15)**	**Ichibor et al. (16)**	**Mangieri et al. (17)**	**Sherwood et al. (18)**	**Munoz-Garcia et al. (19)**
**Selection**								
Representativeness of exposed cohort	※	※	※	※	※	※	※	
Selection of non-exposed cohort	※	※	※	※	※	※	※	
Ascertainment of exposure	※	※		※	※	※	※	
Outcome of interest was not present at start of study	※	※	※	※	※		※	
**Comparability**								
On the basis of the design or analysis	※	※※	※※	※※	※※	※	※※	
**Outcome**								
Assessment of outcome		※	※	※	※	※	※	
Follow-up long enough for outcomes to occur	※	※	※	※	※	※	※	
Adequacy of follow-up	※	※		※	※	※	※	
**Total score**	7	9	7	9	9	7	9	0

### Outcomes

**Figure 3:**
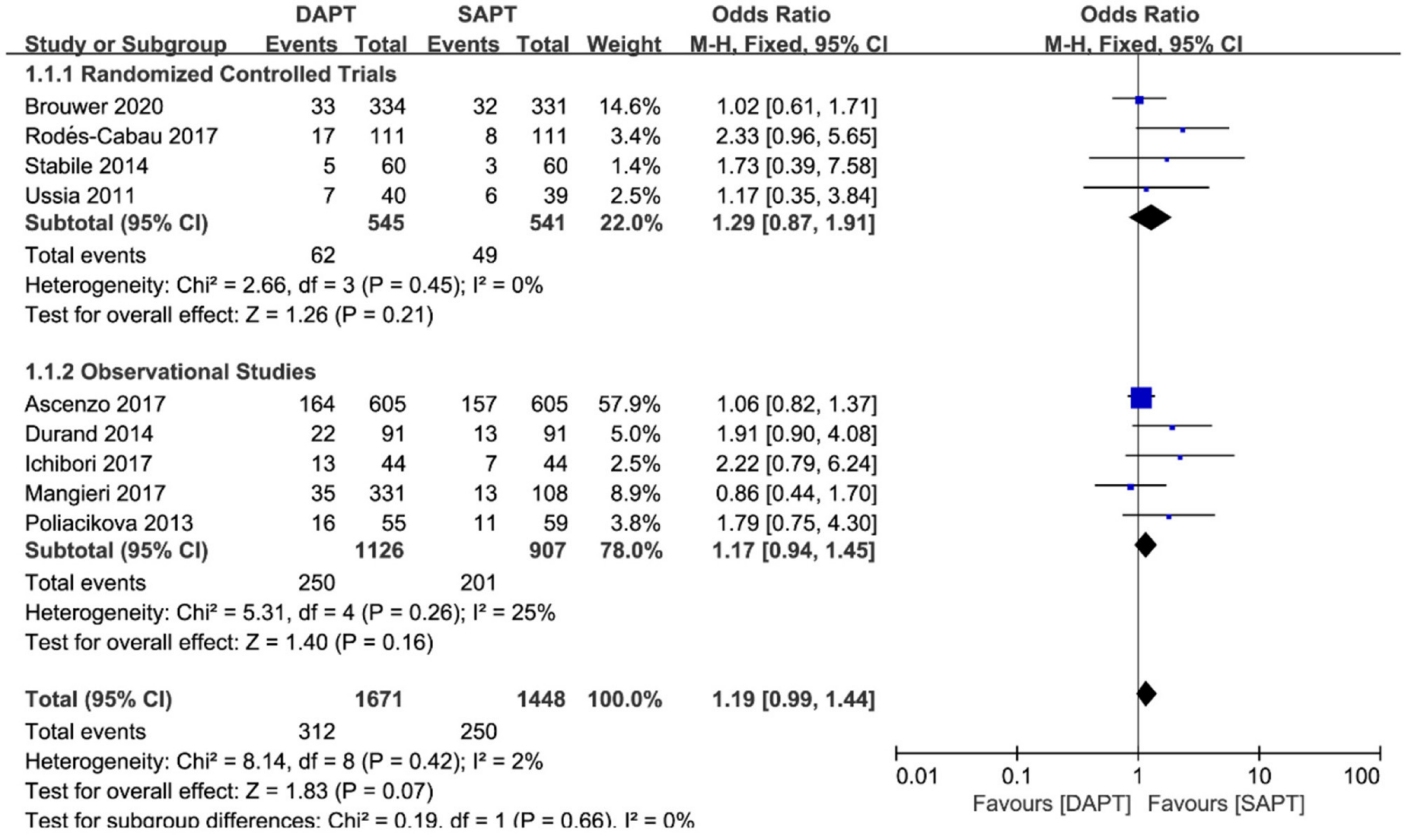
Forest plot for major adverse cardiovascular events (MACE) with subgroup analysis stratified by study type at longest follow-up.

Nine studies (*N* = 3,119) reported MACE data, a composite of cardiovascular death, MI, and stroke. Pooling the data of these studies showed that there was no significant difference but a trend toward higher MACE with DAPT when comparing with SAPT [OR 1.19 (0.99, 1.44), *p* = 0.07] (Figure 3) was evident. Neither stratified by type of study (four RCTs vs. five observational studies) nor sensitivity analysis by removing each individualized study showed a difference in MACE between the two groups.

**Figure 4:**
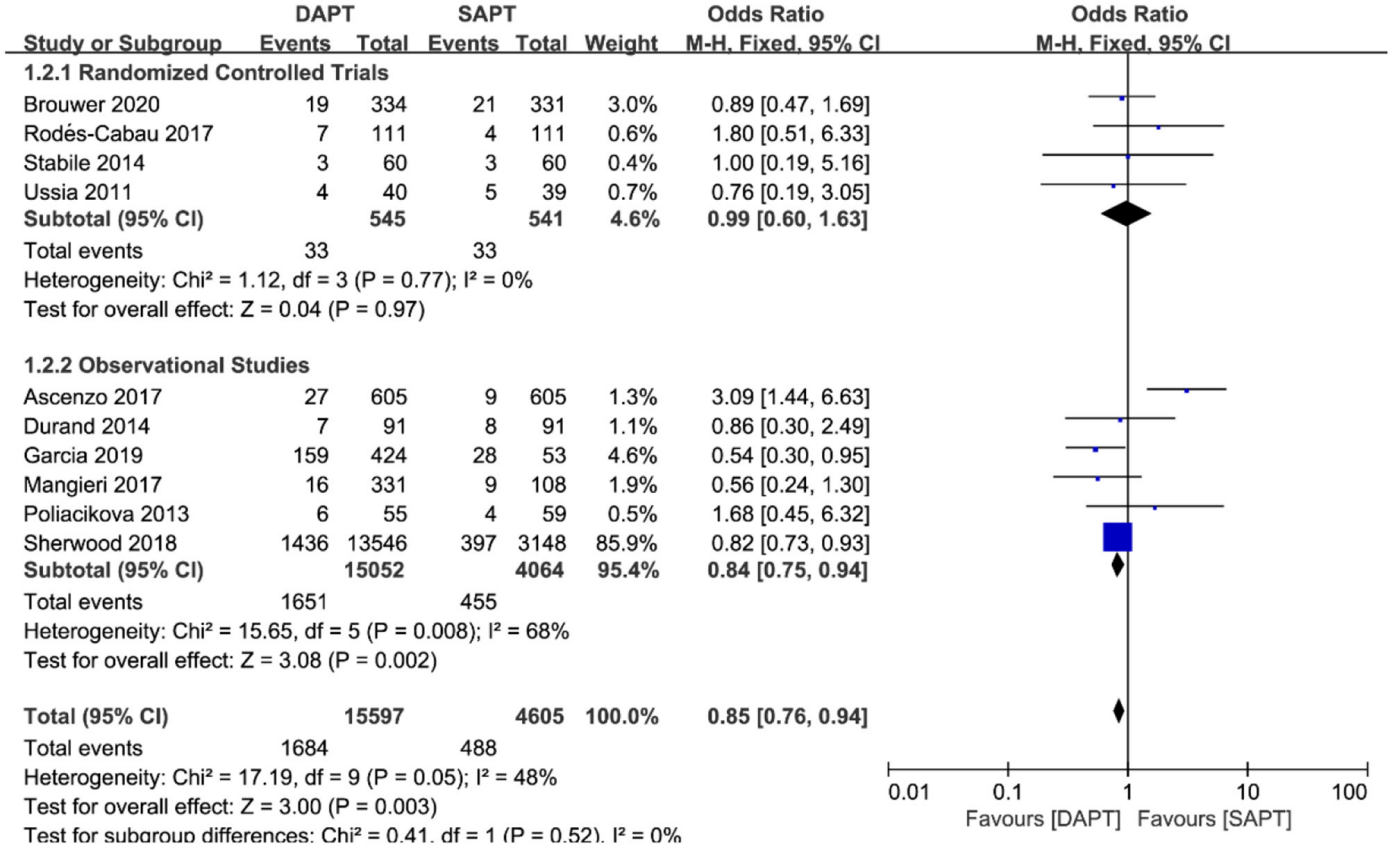
Forest plot for all-cause mortality with subgroup analysis stratified by study type at longest follow-up.

For all-cause mortality in ten studies (*N* = 19,116), a pooled analysis of all studies showed that there was a reduction in the DAPT vs. SAPT group [OR 0.85 (0.76, 0.94), *p* = 0.003] (Figure 4), which was mainly driven by the large weight study of Sherwood et al. (*N* = 16,694). Sensitivity analysis demonstrated a non-statistically significant difference in RCTs or observational studies when removing Sherwood et al. (18). Of note, one study by D'Ascenzo et al. (15) (*N* = 1,210) showed a significant reduction in all-cause mortality in the SAPT group, unlike most other studies.

**Figure 5:**
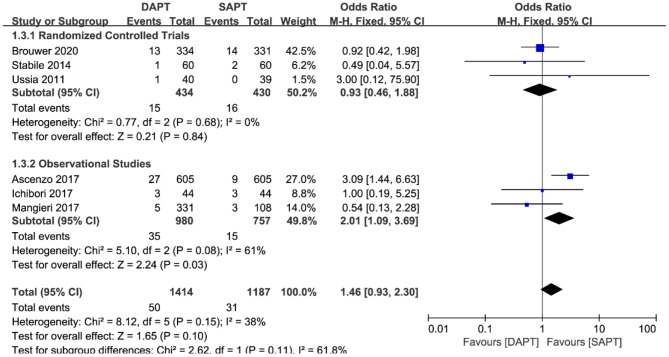
Forest plot for cardiovascular mortality with subgroup analysis stratified by study type at longest follow-up.

There was no difference in cardiovascular mortality by pooling the data of six studies (*N* = 2,601) [OR 1.46 (0.93, 2.30), *p* = 0.10; Figure 5]. When stratified by type of study, there was a significant trend in the SAPT group toward cardiovascular mortality in three observational studies [OR 2.01 (1.09, 3.69), *p* = 0.03), which was not shown in three RCTs. Sensitivity analysis showed that the trend of SAPT was largely driven by D'Ascenzo et al. (15) (*N* = 1,210), and no statistical difference existed when removing it.

**Figure 6:**
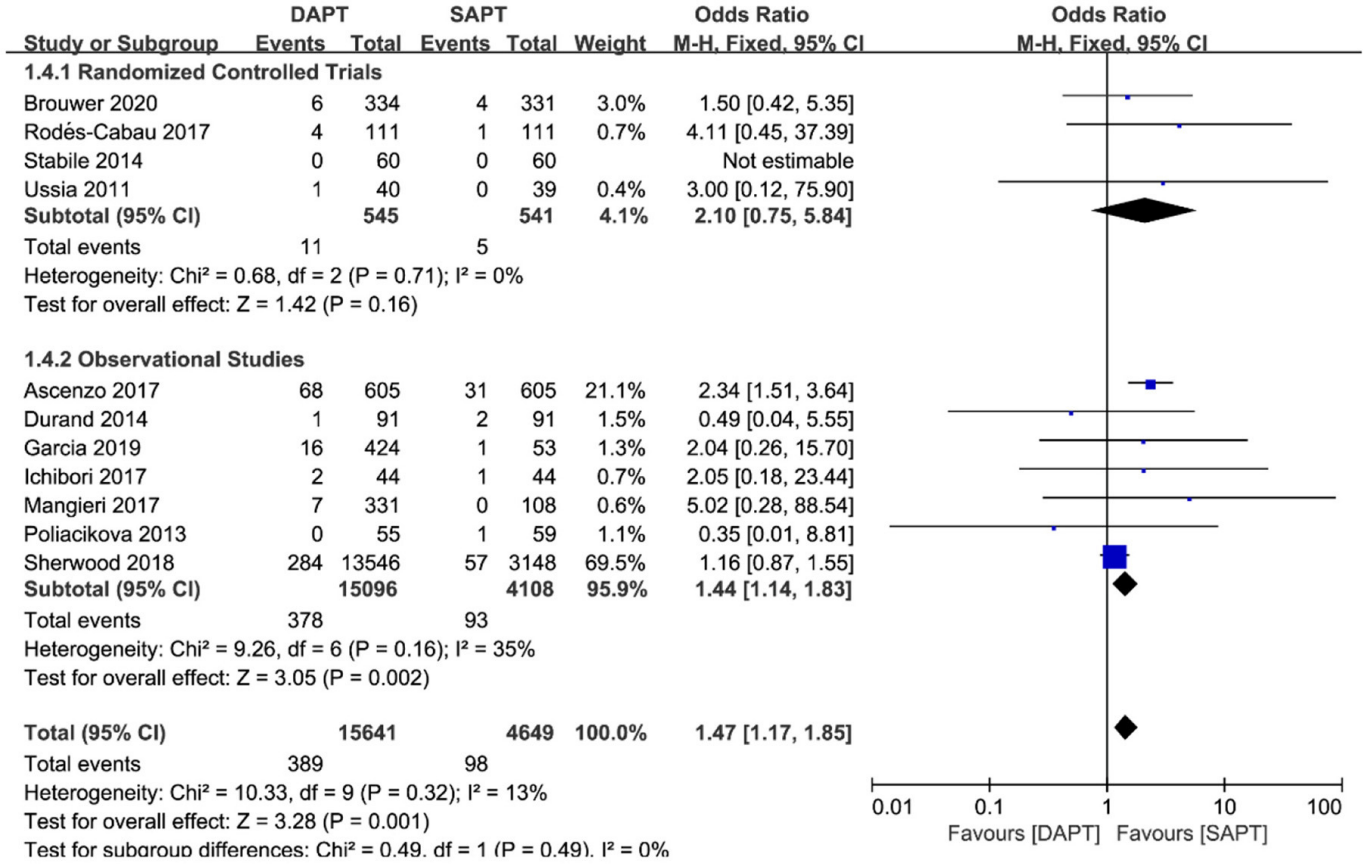
Forest plot for myocardial infarction with subgroup analysis stratified by study type at longest follow-up.

**Figure 7:**
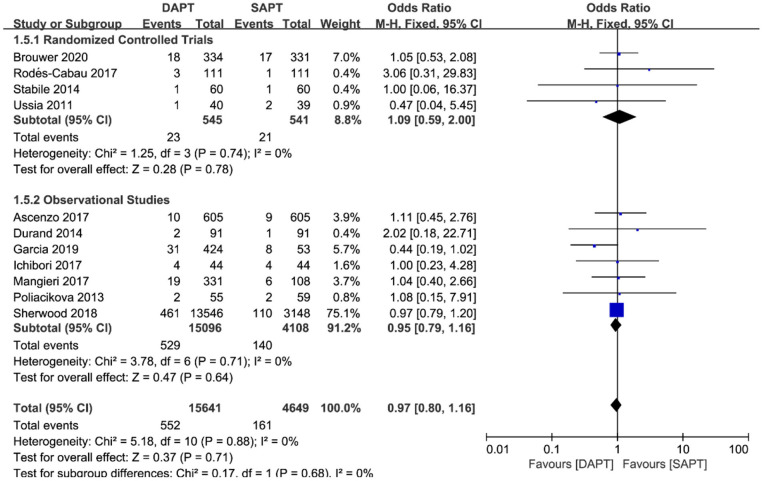
Forest plot for stroke with subgroup analysis stratified by study type at longest follow-up.

As for thromboembolism outcomes, including MI and stroke, MI varied significantly between three RCTs and seven observational studies (*N* = 19,204) (Figure 6). There were no differences between groups in the occurrence of MI events in RCTs [OR 2.10 (0.75, 5.84), *p* = 0.16], but a pooled analysis of observational studies showed a higher risk of MI in the DAPT group [OR 1.44 (1.14, 1.83), *p* = 0.002], which was mainly driven by two studies (15, 18). Interestingly, intensive antiplatelet therapy of DAPT should reduce the incidence of the embolic event of myocardial infarction, whereas the selection bias of the observational study may partly explain the contradictory result. No difference was found in stroke events between the two groups (*N* = 20,290) [OR 0.97 (0.80, 1.16), *p* = 0.71; Figure 7], and the result was of great stability in both subgroup analysis or sensitivity analysis.

**Figure 8:**
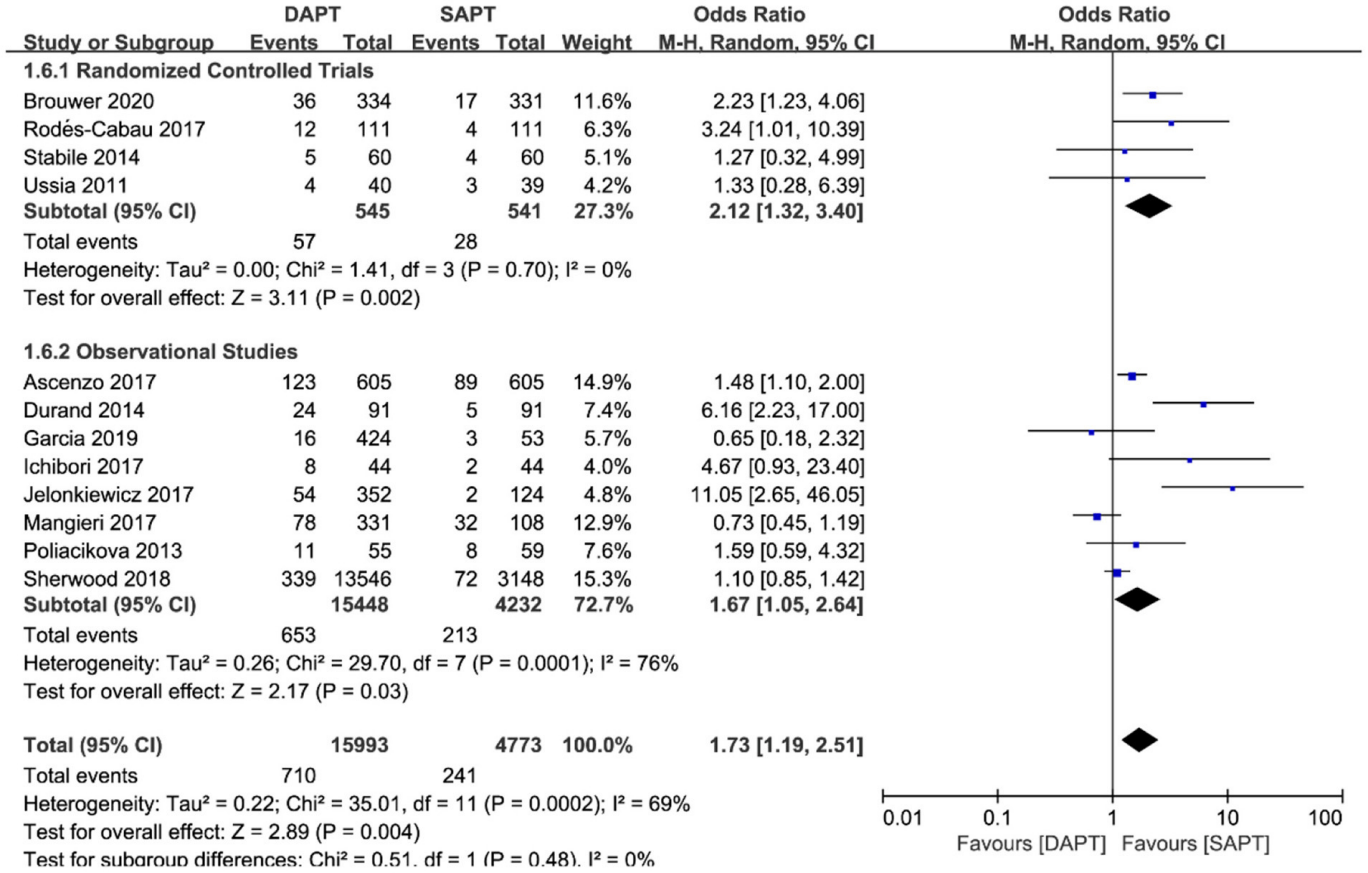
Forest plot for major or life-threatening bleeding (LTB) with subgroup analysis stratified by study type at longest follow-up.

**Figure 9:**
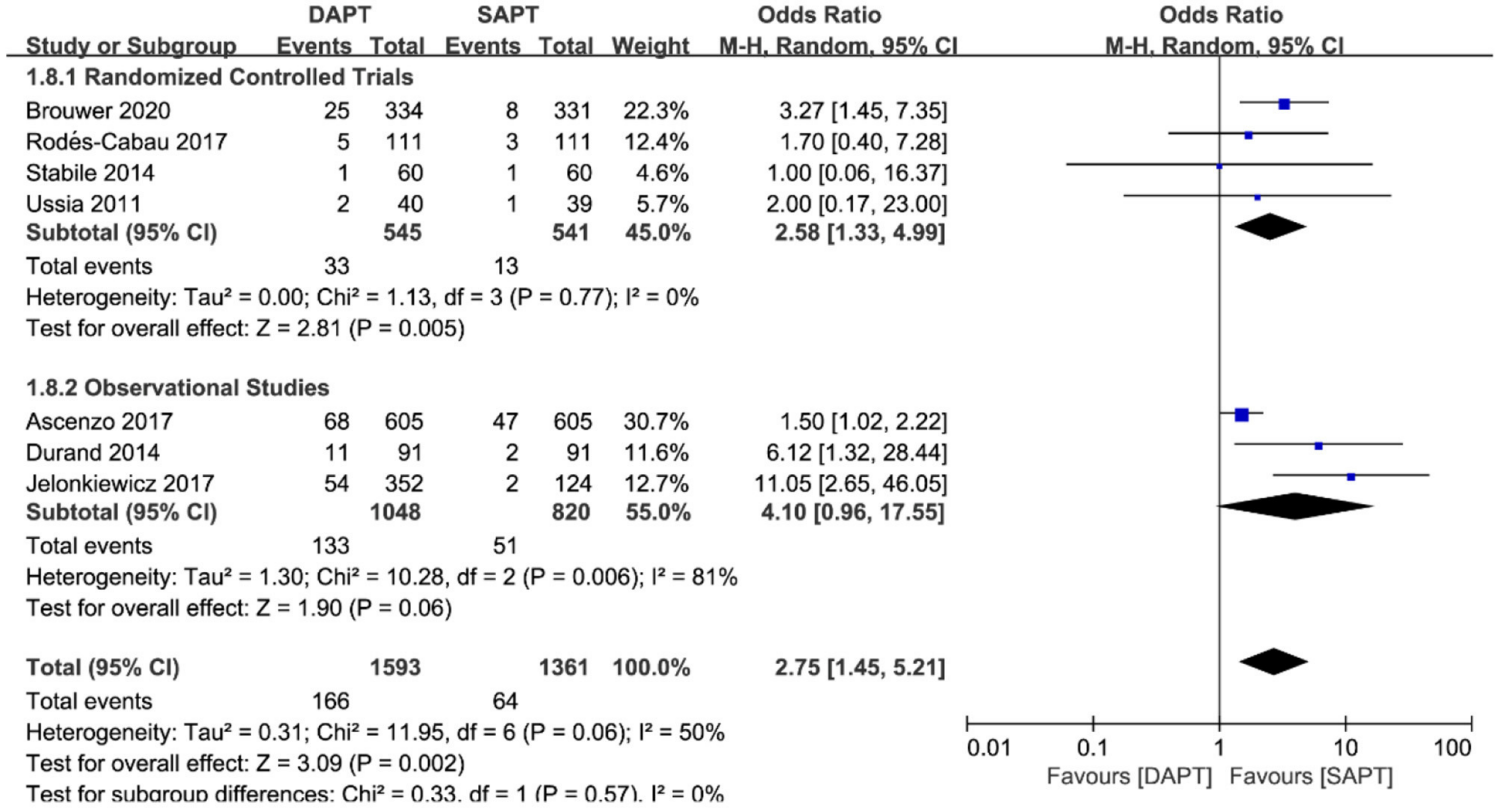
Forest plot for major bleeding with subgroup analysis stratified by study type at longest follow-up.

**Figure 10:**
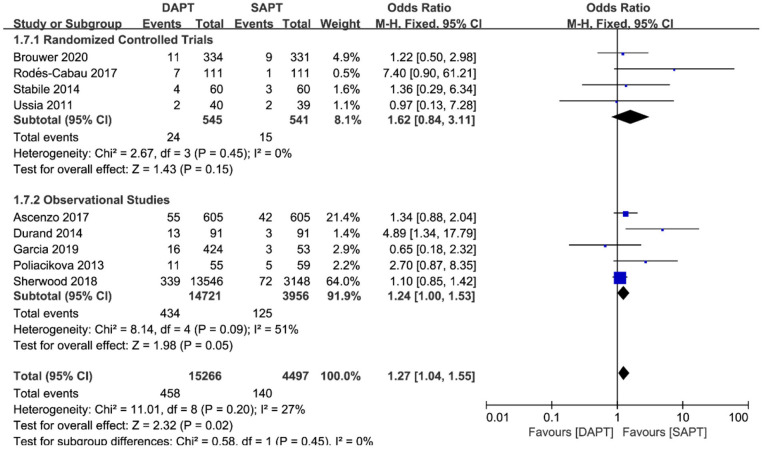
Forest plot for life-threatening bleeding (LTB) with subgroup analysis stratified by study type at longest follow-up.

Combined major and life-threatening bleeding was significantly higher in the DAPT group in both RCTs and observational studies (*N* = 20,766) [OR 1.73 (1.19, 2.51), *p* = 0.004; Figure 8]. The benefits of major vascular complications in the SAPT group were mainly driven by a substantial reduction of major bleeding events [OR 2.75 (1.45, 5.21), *p* = 0.002; Figure 9]. Notably, there were no differences of LTB between the two groups when stratified by type of study, but an increased incidence of LTB was seen when pooling all studies together [OR 1.27 (1.04, 1.55), *p* = 0.02; Figure 10].

## Discussion

In the present meta-analysis, we pooled all eligible studies to compare the safety and efficacy of DAPT vs. SAPT after the TAVR procedure. Our results showed that DAPT post-TAVR was associated with a significantly increased risk of major or life-threatening bleeding and was non-superior to SAPT in terms of ischemic events of MI and stroke. Furthermore, there was no significant difference in MACE, all-cause mortality, and cardiovascular mortality between the two groups. Thus, these data lend support to SAPT as an optimal antithrombotic strategy following TAVR, which can improve the prognosis of patients.

The pooling data of all studies indicate that there existed no significant difference in MACE, all-cause death, and cardiovascular death comparing DAPT with SAPT after TAVR. However, further subgroup analysis and sensitivity analysis often showed inconsistent results, mostly due to high-weight observational studies. The retrospective nature of observational study developed its inevitable bias. Physicians tend to make antiplatelet strategies based on empirical assessment of patients' bleeding and ischemic risk profile. For instance, patients were more likely to be assigned to the DAPT group because of MI or PCI history and the SAPT group because of relative higher bleeding risk. Hence, great baseline heterogeneity between the two study groups in the observational studies leads to controversial results, as previous meta-analyses have revealed (20, 21). Take all-cause death for example, DAPT showed no difference compared with SAPT in RCTs but had favorable effects in observational studies. However, these benefits disappeared when the large powered study by Sherwood et al. (18) was removed from sensitivity analysis. When analyzing reasons for inconsistent results of all-cause death among studies, we noted that a substantial proportion of the all-cause deaths were non-cardiovascular deaths due to malignant diseases and comorbid chronic conditions, which were irrelevant to antiplatelet strategies. For cardiovascular death, the SAPT group showed lower mortality than DAPT in observational studies and no difference in RCTs. That is, despite contradictory results in these events, SAPT was always safe and non-inferior to DAPT.

The current guideline recommends DAPT treatment for the first 3–6 months after TAVI, a temporary intensified antiplatelet regimen is considered to mitigate thromboembolization risk mediated by prosthetic valve before the valve endothelialization has been completed. Nevertheless, our results showed that DAPT was not associated with reducing myocardial infarction and stroke events. Especially for stroke, both subgroup analysis and sensitivity analysis showed constant differences between the two groups. For myocardial infarction events, SAPT even showed superior results in observational studies, although such a difference disappeared when the large weight studies were removed (15). Periprocedural ischemic complications were usually caused by embolization of acute device-related thrombus, atheroma, calcium, or connective tissue debris developed with mechanical manipulation and new-onset atrial fibrillation (22). Hemodynamic instability and hypoperfusion during rapid ventricular pacing in TAVR may also lead to stroke. The turbulence caused by malposition and mismatching of the anatomical structure and the bioprosthetic valve may increase thrombotic risks, partially explaining the inconsistent results of embolism risk in subgroup analysis between the two groups. Post-TAVR ischemic events mostly occurred during the hospital stay, ~50% within 24 h of the procedure (23). Therefore, an intense antithrombotic strategy with DAPT has faint benefits on embolism events; prolonged DAPT for 3–6 months after discharge appears unnecessary. An individualized antithrombotic strategy that comprehensively integrates the characteristics of aortic valvular diseases, devices, procedures, and post-TAVR flow dynamics may have a more important role. Regarding the prosthetic valve's endothelialization for integration into the aortic wall, studies have found that SAPT can also promote the completion of endothelialization without prolonging the duration of antiplatelet therapy (24). Subclinical valve thrombosis considered with increased risk of stroke, though rare, can be detected with 4-dimensional computed tomography, according to which physicians could develop an individualized antithrombotic strategy of transition to a more aggressive antiplatelet strategy during follow-up (25).

Intensifying the antiplatelet regimen with DAPT comes at the cost of increased major and LTB events, largely driven by major bleeding events. In terms of LTB events alone, there was no difference between the two groups in each subgroup analysis, but the pooling data of all eligible studies suggested an increased risk of LTB. For the primary driver of benefit in bleeding events, high-quality RCTs found that DAPT significantly increased the risk of major bleeding, and there were still consistent differences in the pooled analysis. The bleeding events did not reach a statistical difference in the observational studies because of the inherent limitation of observational design, in which the SAPT group was mostly the population with high bleed risk, while the DAPT group had lower bleeding incidence. The recommended DAPT treatment after TAVR was based on expert consensus, though purely empirical, and was developed from abundant practical experience on antithrombotic strategies after PCI, with consideration of the prosthesis endothelialization and requirement of reducing thromboembolism. However, with the increasing number of TAVR procedures, the current incidence of stroke events after TAVR is far less than that of bleeding events (26). Therefore, more attention has been paid to searching for an optimal antiplatelet strategy to reduce bleeding on the premise of no increased thromboembolism. For example, bleeding has served as the primary endpoint in the POPular-TAVI trial (11). Increased bleeding risk in the DAPT group was mainly attributed to the fact that TAVR candidates were usually elderly with a high-risk profile of bleeding and tended to have multiple comorbid illnesses with a bleeding tendency such as chronic kidney disease, anemia, and gastrointestinal disease. Acute postoperative bleeding of TAVR was mostly attributed to the procedure itself rather than the antiplatelet regimen, commonly seen at the location of vascular access. Such bleeding was associated with large-bore catheters, frail vessels due to peripheral vascular diseases, inappropriate anticoagulation during the procedure, and transapical access (27). Major bleeding occurring after TAVR increases within 24 h, peaks at 7 days, mostly within 1 month postoperatively, and sometimes evolves into LTB because of no timely detection. Thus, efforts to reduce bleeding events after TAVR require integrated management, comprised of an optimal antiplatelet strategy, novel advanced devices, more skilled operators (28), meticulous postoperative monitoring, and nursing, together ultimately improving the prognosis in patients undergoing TAVR.

## Conclusion

As an updated meta-analysis pooling maximum studies of eligibility up to now, our results demonstrate that, compared with SAPT, post-TAVR DAPT is associated with a significantly increased incidence of major or life-threatening bleeding without additional benefits in preventing thrombotic events such as MI and stroke. Although further studies are warranted to establish the optimal antithrombotic regimen, SAPT is promising to serve as the first recommended antiplatelet strategy in patients undergoing TAVR without indication for anticoagulants when guidelines are updated in the next version.

## Data Availability Statement

The original contributions generated for the study are included in the article/supplementary material, further inquiries can be directed to the corresponding author/s.

## Author Contributions

BH and YZ proposed the idea for the study and finished the study design. YZ and WY retrieved studies, collected and extracted data with disagreements resolved by LS and BH. YZ performed the meta-analysis and drafted the manuscript with a complete review by LS and BH. All authors read and approved the final manuscript.

## Conflict of Interest

The authors declare that the research was conducted in the absence of any commercial or financial relationships that could be construed as a potential conflict of interest.
